# What Is Eczema?

**Published:** 1898-10-29

**Authors:** 


### WHAT IS ECZEMA?

It seems impossible at the present moment to give a satisfactory answer to this question, or perhaps it would be more correct to say an answer which will be satisfactory to all parties. Mr. Malcolm Morris says that we "know a great deal about eczema, but we are not yet agreed as to what eczema is." Nevertheless, although John Hunter was well within the mark in saying that "definitions are the most damnable things," they still are necessary evils, and we must accept with due thankfulness any careful attempt to compress large facts into short phrases. Mr. Morris says that his conception of eczema is expressed in the following definition: "A catarrhal inflammation of the skin, originating without visible external cause irritation, and characterised in some stages of its evolution by serous exudation." It is to be noted that in this definition he excludes such cases of what we suppose we may call catarrhal dermatitis as are caused by chemical or mechanical irritation of the surface, although on the parasitic theory it does not seem absolutely clear why disturbance of the integrity of the tissues from without should not predispose to microbic growth just as much as disturbance from within. Putting this on one side, however, and admitting that parasitism has much to do with the causation of eczema, although in itself generally insufficient to cause the disease, and taking the supplementary factor as being some nerve disturbance, Mr. Morris answers the question, What are we to understand by eczema? broadly as follows: "It is a disease the most striking clinical character of which is the infinite variety of lesions by which it displays itself; originating in the action of parasites on a skin the resistance of which has been enfeebled by pre-existing disease or structural abnormality or by disordered innervation; sometimes made more intractable by gout and other constitutional states, but having no direct relation to the general health." Perhaps the most interesting part of this conclusion lies in what is said about eczema having no direct relation to the general health. The position Mr. Morris takes up is stated in the following sentence: "As for gout, diabetes, Bright's disease, and chronic dyspepsia, my experience is that in relation to eczema they are 'aggravating circumstances' and nothing more." Here again so much depends on definitions. We are quite ready to admit that gout is but an aggravating circumstance, but when a circumstance is so aggravating as to turn what may have existed for months in the form of a slight papular eruption into an irritating, sleep-destroying dermatitis, we may well question whether gout and dyspepsia, to take two leading examples of states of "general health," do not deserve a more exalted position in the etiology of eczema than that of "aggravating circumstances."

